# Vegetation Controls on Weathering Intensity during the Last Deglacial Transition in Southeast Africa

**DOI:** 10.1371/journal.pone.0112855

**Published:** 2014-11-18

**Authors:** Sarah J. Ivory, Michael M. McGlue, Geoffrey S. Ellis, Anne-Marie Lézine, Andrew S. Cohen, Annie Vincens

**Affiliations:** 1 Brown University, Providence, Rhode Island, United States of America; 2 University of Kentucky, Lexington, Kentucky, United States of America; 3 U.S. Geological Survey, Denver, Colorado, United States of America; 4 LOCEAN, CNRS, Paris, France; 5 University of Arizona, Tucson, Arizona, United States of America; 6 CEREGE, CNRS, Aix-en-Provence, France; Université Pierre et Marie Curie, France

## Abstract

Tropical climate is rapidly changing, but the effects of these changes on the geosphere are unknown, despite a likelihood of climatically-induced changes on weathering and erosion. The lack of long, continuous paleo-records prevents an examination of terrestrial responses to climate change with sufficient detail to answer questions about how systems behaved in the past and may alter in the future. We use high-resolution records of pollen, clay mineralogy, and particle size from a drill core from Lake Malawi, southeast Africa, to examine atmosphere-biosphere-geosphere interactions during the last deglaciation (∼18–9 ka), a period of dramatic temperature and hydrologic changes. The results demonstrate that climatic controls on Lake Malawi vegetation are critically important to weathering processes and erosion patterns during the deglaciation. At 18 ka, afromontane forests dominated but were progressively replaced by tropical seasonal forest, as summer rainfall increased. Despite indication of decreased rainfall, drought-intolerant forest persisted through the Younger Dryas (YD) resulting from a shorter dry season. Following the YD, an intensified summer monsoon and increased rainfall seasonality were coeval with forest decline and expansion of drought-tolerant miombo woodland. Clay minerals closely track the vegetation record, with high ratios of kaolinite to smectite (K/S) indicating heavy leaching when forest predominates, despite variable rainfall. In the early Holocene, when rainfall and temperature increased (effective moisture remained low), open woodlands expansion resulted in decreased K/S, suggesting a reduction in chemical weathering intensity. Terrigenous sediment mass accumulation rates also increased, suggesting critical linkages among open vegetation and erosion during intervals of enhanced summer rainfall. This study shows a strong, direct influence of vegetation composition on weathering intensity in the tropics. As climate change will likely impact this interplay between the biosphere and geosphere, tropical landscape change could lead to deleterious effects on soil and water quality in regions with little infrastructure for mitigation.

## Introduction

Tropical climate is rapidly changing resulting in altered atmospheric and oceanic circulation, as well as changing variability of important climatic modes like the El Niño–South Oscillation [1]. Much work is being done within the framework of the IPCC to better understand climate sensitivity and climate-induced changes to the biosphere; however, integrating the effects on and feedbacks from the geosphere has been largely unexplored [2]. Alterations to the geosphere, an important component of the Earth's critical zone, could have costly, hazardous implications for water and soil quality, as well as a host of ecosystems services provided by inland waters (e.g., serving as fisheries, rookeries, and zones of stormwater retention).

Weathering and climate have hypothetically been linked via feedback loops that regulate the Earth system [3]. However, the low resolution of most marine sedimentary records and the strong influence of long time-scale (10^6^–10^7^ yrs) processes such as orogeny do not provide a scalable framework for evaluating future changes in the critical zone. Additionally, although climate has long been thought to play a strong, direct role, a number of datasets indicate that the role of climate for weathering and erosion may in fact be indirect, mediated by vegetation and soil storage of organic acids [4]–[7].

Some studies have generated geochemical indicator records of weathering or produced denudation rates for tropical watersheds based on mass balance models; however, such studies are unable to determine how these rates vary over long time-scales [8]–[11]. Due to the strong interrelation of vegetation and climate, it has been particularly difficult to separate the individual influences of each mechanism. However, it is expected that vegetation plays an important role. Other studies that have examined the relationship between land use and sediment yield in tropical catchments have demonstrated that heavily forested areas generate less sediment than grasslands or areas modified for agriculture or ranching (e.g., [12]). Furthermore, human land-use change could have strong but unclear effects on weathering and the carbon cycle via alteration of vegetation with no climatic change [13]. To answer the question of how climate change influences weathering and erosion in the tropics, well-dated, high-resolution paleo-records from the critical zone are needed. However, records of tropical lowland vegetation and climate are exceedingly rare, despite the great need to better understand these regions and their growing populations, as articulated by the IPCC [1]. Existing Quaternary paleoenvironmental records from tropical Africa attest to the fact that both vegetation and climate have varied greatly over the last 20 ka [14]. However, it is as yet uncertain what the relative importance of their effects on the geosphere may have been.

**Figure 1:**
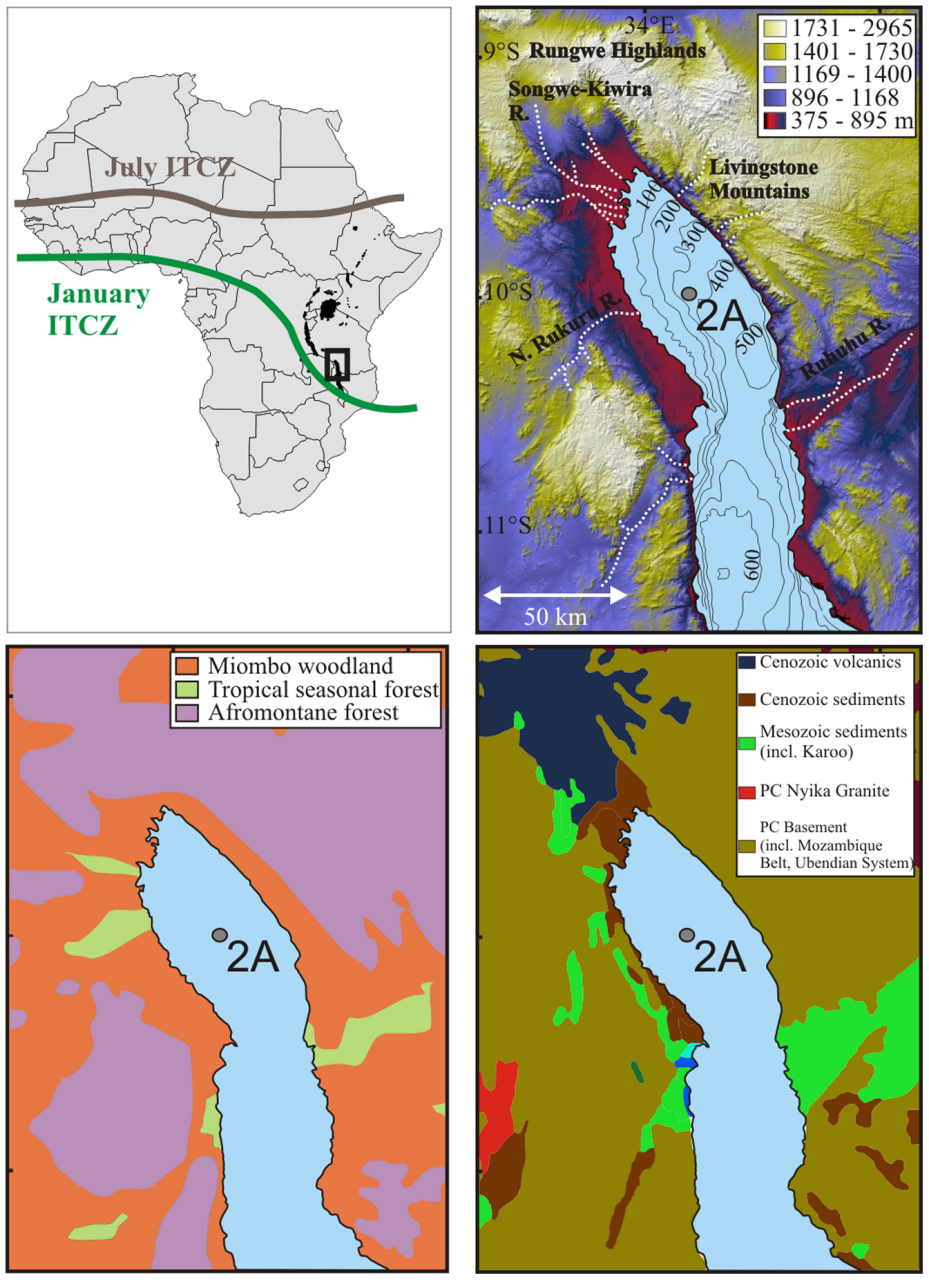
Geography, vegetation, and geology of the study site. (A) showing the July and January positions of the Intertropical Convergence Zone (ITCZ); rectangle represents inset area for watershed maps (modified from Nicholson, 1996). (B) map of the northern basin of Lake Malawi showing topography and bathymetry (isobaths [in meters] modified from Scholz et al., 1989), (C) modern potential vegetation distribution (modified from White, 1983), and (D) bedrock geology (modified from Schlüter, 2006). Scale for panels C and D is the same as for panel B. 2A identifies the location of drill core MAL05-2A.

Our study seeks to address this important knowledge gap using a scientific drill core from Lake Malawi, southeast Africa (Figure 1). The lake is situated at the current southern extent of the Intertropical Convergence Zone (ITCZ) and has been found to be climatically and ecologically sensitive, making it a remarkable natural laboratory to investigate the relationships and feedbacks among vegetation, climate, weathering, and erosion during the last deglaciation (18–9 ka). This period is of interest because of high-amplitude changes in both climate and vegetation that are both progressive and abrupt. This record thus has the potential to assess the influence of climate change on weathering in the tropics.

## Modern Setting

Lake Malawi is the southernmost rift lake in the western branch of the East African Rift System (Figure 1). The physical geography of Lake Malawi and its watershed is controlled by Cenozoic extensional tectonics and volcanism [15]. The lake occupies a series of half-graben basins that are linked *en echelon*, such that steeply dipping border faults with opposing polarity are connected by accommodation zones [16]. Subsidence and deformation patterns within individual half-graben basins control lake bathymetry, with maximum depths (∼700 m) achieved adjacent to border faults [17].

The drill core used in this study was collected from the northern basin of the lake (Figure 1). Onshore, distinct topography and drainage patterns follow major tectonic features. A border fault margin to the east of the lake is characterized by high-elevation escarpments which form the Livingstone Mountains. The topography of this faulted margin precludes all but short, steep river systems from forming [18]. In contrast, flexural margins and accommodation zones exhibit comparatively lower altitudes and relief, and large river systems are common in these settings [19]. North of the lake, a well-developed axial delta system building along low-gradient plains is sourced by the Songwe-Kiwira River, which drains the Rungwe highlands. Because of the sloping topography and presence of the delta, Johnson and McCave [20] considered the Songwe-Kiriwa River to be the only significant sediment source to the northern basin. The Songwe-Kiriwa delta has a relatively gentle subaqueous slope (1∶70) with a broad shelf, reaching 2–3 km offshore [19]. The drill core site is located ∼40 km from the mouth of the river. Although the nearest shoreline is 20 km from the drill site, a sub-lacustrine trough to the east probably prevents significant clastic input from the eastern part of the watershed [20]. Offshore, the lake deepens adjacent to the border fault to ∼500 m, and sedimentary processes are dominated by debris and turbidity flows [21].

The smaller watersheds that drain into the lake consist primarily of Neogene alkaline volcanic bedrock, with abundant olivine and alkali basalts, phonolites, trachytes, and nephelinites [22]. Permo-Triassic and Cretaceous sedimentary rocks also crop out north and west of the lake (Figure 1). Soils in the woodlands and forests of the lowlands dominantly consist of pellic vertisols and mollic andosols [23]. The higher-gradient watersheds along the northeastern margin of the basin draining the Livingstone Mountains consist primarily of the Neoproterozoic Mozambique belt, with abundant biotite-horneblende-pyroxene gneiss, charnockites, and minor schists and quartzites [22]. Soils in this area are typically thin and weakly developed, consisting of lithosols, chromic cambisols and dystric regosols [23].

Climate within the watershed is primarily controlled by the yearly passage of the ITCZ, imparting a highly seasonal rainfall regime in which rainfall occurs from November to March. A single, long dry season lasts from April to October when little to no rainfall occurs and prevailing winds are strong southeasterlies [24]. Mean annual precipitation varies from ∼800 mm/yr in the lowlands to over 2400 mm/yr in the Rungwe highlands [25]. In addition to a marked N-S gradient of precipitation, the rift escarpments bordering the lake create a pronounced local orographic effect. This results in substantial variability in local rainfall over relatively short spatial scales throughout the watershed.

Vegetation within the watershed is controlled by rainfall and dry season length, although temperature plays a role in the subalpine and alpine zones of the highlands [26]–[28]. Four principal biome types are observed within the Malawi watershed. In the lowlands (<1500 masl), Zambezian miombo woodlands, a low-diversity deciduous tropical woodland, dominate the landscape. These woodlands primarily comprise *Uapaca*, *Brachystegia*, *Isoberlinia*, *Julbernardia*, and species of Combretaceae and are relatively open, with 30–70% canopy cover. Tropical seasonal forests, closed-canopy semi-deciduous forests— with trees such as *Myrica*, *Macaranga*, Ulmaceae, and Moraceae—are not common in the lowlands; however, they are typically found in areas with locally moist conditions and as riparian corridors along streams and rivers. In the highlands (>1500 masl), afromontane vegetation is found. Today, this region consists of discontinuous patches of afromontane forest separated by high-elevation grasslands. Within the forests, composition is controlled by elevation and rainfall with lower montane forests having moister forest taxa such as *Olea capensis* (1500–2000 mm/yr; 0–3 dry months) from 1500 to 2500 m, whereas *Podocarpus*, *Juniperus*, Ericaceae are common above 2000 m in drier sites (800–1700 mm/yr; ∼4 dry months) [27]. The most open biome occurs in the southernmost part of the watershed, where rainfall is the lowest (<800 mm/yr) and contains wooded grasslands with Zambezian affinities.

## Methods

**Figure 2:**
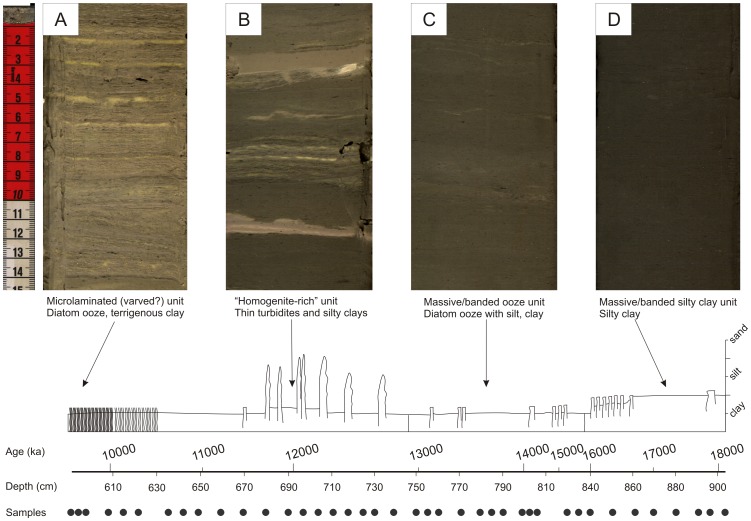
Core MAL05-2A lithostratigraphy within the study interval. Sediment composition and structures vary significantly over the deglacial interval.

Core MAL05-2A was collected from the northern basin of Lake Malawi (10°1.1′S, 34°11.2′E; 359 m water depth) during the Lake Malawi Drilling Project (LMDP) in 2005 (permits issued by Malawi Geological Survey) [29]. Coring site, stratigraphy, and age model details can be found in Figures 1 and 2 as well as in Brown et al. [11] and Scholz et al. [29]. We studied a total of 40 samples, which were taken every ∼5 cm from a 3-meter core section (∼6–9 meters below lake floor [mblf]). The only gap occurred at ∼8.1 mblf, where not enough sediment was available in the core archive to maintain the routine sample interval. The age model of the upper 22 m of the core is based on 24 calibrated accelerator mass spectrometry ^14^C dates fit with a second-order polynomial (calibrated with the “Fairbanks 0107” calibration curve [30] so as to make ages comparable to those of previous studies). The average temporal resolution within the studied section is 208 years, but is ∼100 years from 14–11 ka.

Terrigenous mass accumulation rates (TMAR) are a metric of the amount of sediment entering the lake from the watershed and provide insights on the erosion of Lake Malawi's northern watershed. Calculation of TMAR relied on several datasets. Linear sedimentation rates (cm/yr) were calculated using the radiocarbon age model presented in earlier LMDP publications [11]. Dry bulk density was calculated for MAL05-2A sediment horizons using the formulas presented in Dadey et al. [31]. We calculated water content for this purpose and used the gamma-ray density curves from GEOTEK multi-sensor logging of the core [32]. Sediment mass accumulation rates (g/cm^2^/yr) were calculated by multiplying the linear sedimentation rate and dry bulk density. Weight percent terrigenous sediment was determined from X-ray patterns analyzed using the RockJock quantitative mineralogy computer program [33].

Terrigenous particle size data (sand: >62.5 µm; silt: 3.9–62.5 µm; clay: <3.9 µm), interpreted in conjunction with core lithostratigraphy, provide an indicator of landscape sediment flushing, sub-lacustrine hydrodynamic energy, and depositional processes [e.g., [34]]. Particle size analysis was conducted on the terrigenous fraction of the core MAL05-2A samples using a Malvern laser-diffraction particle size analyzer coupled to a Hydro 2000S dispersion bench (see Methods S1).

Detrital clay minerals in lake sediments provide evidence for the alteration of watershed parent lithologies by physical (disintegration) and chemical (compositional alteration) weathering processes. Quantitative mineralogy was determined using powder X-ray diffraction (XRD) and the computer program RockJock v.11, which has been successfully used for the analysis of fine-grained Cenozoic sediments at a number of locales globally (e.g., [35]–[37]). For more information on the preparation and processing of these samples, see Methods S1.

In our interpretation of clay mineralogy, we focus on the ratio of kaolinite to smectite (K/S) as a proxy for chemical weathering intensity, which has been used in other similar studies in Africa [38]–[39]. Kaolinite is produced by the process of leaching in tropical regions marked by high rainfall [40]. In contrast, smectite is typical of tropical semiarid regions and points to increased rainfall seasonality, as it frequently forms during the dry season from the concentration of chemical elements transported to downstream areas by runoff [41]–[43]. We interpret elevated percentages of micas (illite plus chlorite) to reflect soil formation influenced by physical weathering processes [44].

Pollen samples were taken at the same depths as those for particle size and clay mineralogy and were processed following the standard methods of Faegri and Iverson [45]. More details about sample preparation and pollen identification can be found in Ivory et al. [46]. Vegetation groupings presented in this study are based on biomes of White [27], as well as prior pollen studies within the watershed by Debusk [25] and Vincens et al. [47].

## Results

### Vegetation

**Figure 3:**
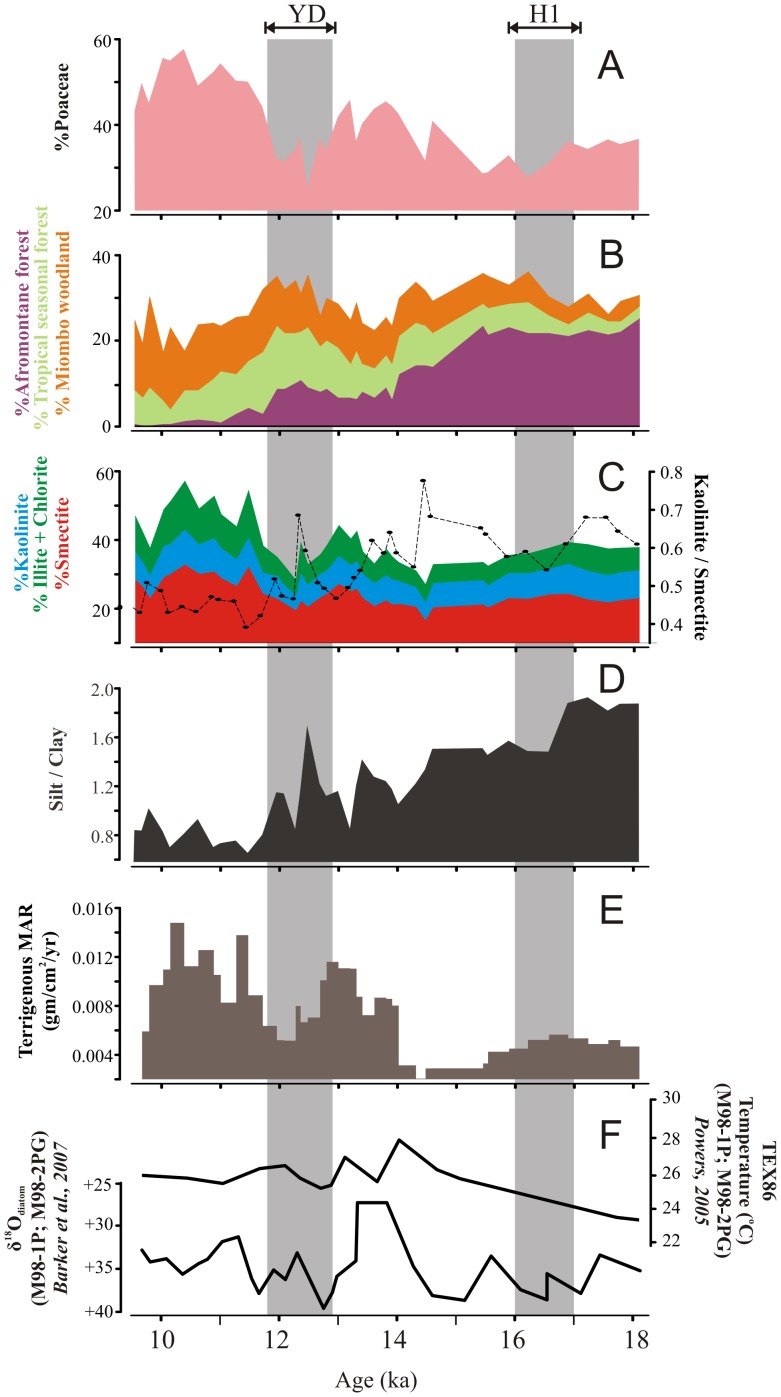
Vegetation and weathering indicators from drill core MAL05-2A.YD, Younger Dryas. H1, Heinrich event 1. (A) pollen percentages of Poaceae and (B) arboreal vegetation types (afromontane forest, tropical seasonal forest, miombo woodland), (C) XRD clay mineral percentages of smectite, kaolinite, and illite+chlorite, ratio of kaolinite to smectite (K/S), (D) ratio of silt to clay (silt/clay), and (E) terrigenous mass accumulation rate, and (F) δ^18^O from diatoms and TEX-86 temperature from piston cores M98-1P and M98-2PG from the northern basin of Lake Malawi from Barker et al. (2007) and Powers (2005), respectively.

Detailed descriptions of the ecological dynamics surrounding vegetation change in the Malawi watershed during the last deglaciation appear in Ivory et al. [46]. In this study, we present pollen taxa grouped into biomes (afromontane forest, tropical seasonal forest, Zambezian miombo woodland) in order to present the composition and physiognomy of vegetation on the landscape over time. Following the Last Glacial Maximum (LGM) at 18.1–16.4 ka, afromontane forest taxa percentages were relatively stable at around 20% (Figure 3). Their subsequent decline began in a stepwise manner until 14 ka, at which time, the high-elevation arboreal taxa stabilized at percentages of 7 to 10%. Coeval with the decline of montane taxa, percentages of both lowland arboreal vegetation types increased. Beginning at 14 ka, both tropical seasonal forest and miombo woodland increased progressively in abundance until ∼11.8 ka from percentages of <5% up to ∼13%. Similarly, a progressive increase in grasses is observed in the record from around 30% to over 45% following the decline of afromontane forest; however, grasses once again returned to lower values around 30% relatively abruptly at 13 ka before the increase of the lowland arboreal vegetation types. The lowland arboreal vegetation continued to increase in a similar manner to a maximum value of 13.5% until 11.8 ka when trends in the tropical seasonal forest and miombo woodland diverged. Forest taxa began a slow decline until a minimum of 3.5 to 7% at 10 ka. At this same time, grasses began to increase at the expense of lowland trees with abundances of nearly 60% by 11 ka. Miombo woodland continued to increase until the end of the record at a maximum value of around 15%.

### Mineralogy and Particle Size

Qualitative analysis of X-ray diffraction patterns generated from oriented clay mounts indicated the presence of smectite, kaolinite, illite, and minor chlorite in MAL05-2A (for more information, see the Methods S1). These results are in accord with the findings of Kalindekafe et al. [48] and Branchu et al. [49] for the modern clay mineralogy of northern Lake Malawi. Using RockJock, we determined that smectite was the most abundant clay in the deglacial sequence at all times, ranging from 9 to 29%. Kaolinite ranges from 6 to 13%, and illite ranges from 4 to15%. At the end of the LGM, kaolinite:smectite (K/S) within the lake sediments is relatively high, with values varying from 0.55 to 0.70 from 18.1 to 15 ka. The maximum value (0.79) of K/S within the lake is reached at 14.5 ka. After this time, sediment K/S begins a two-step decline with values of 0.55 to 0.65 from 14.5 to 13 ka, followed by highly variable values from 0.45 to 0.70 from 13 to 11.8 ka. It is only at the onset of the early Holocene at 11.8 ka that stable minimum values are reached (0.40 to 0.50). In addition, illite plus chlorite (maximum  = 18%) increased sharply for the first time in the record at 11.8 ka and remained at stable values for the rest of the record.

Over the entire record (∼18.1 to 9.5 ka), there is a decreasing trend in the ratio of silt to clay (silt/clay) which is marked by a two-step transition towards finer material. In the interval from ∼18.1 to 14.6 ka, we observe high relative silt/clay values, with a mean of 1.69. Here, particles in the silt-size class (∼4.0 to 62.5 µm) dominated the terrigenous fraction. The form of the silt/clay curve is blocky during this early interval, with prolonged periods of invariant conditions on the order of 1000–2000 years in duration. Values of silt/clay from ∼18.1 to 16.9 ka range from ∼1.84 to 1.95, whereas values from ∼16.9 to 14.6 ka range from 1.48 to 1.59. A transition in the record after ∼14.6 ka is marked by the saw-tooth form of the silt/clay curve until ∼11.9 ka. During this more variable interval, values of silt/clay range from 0.87 to 1.72 with a mean of 1.21. The highest silt/clay values in this interval correlate with high relative values of sand (up to ∼3.0%); this was the only instance very fine sand was detected by our analysis. The second important transition in the record took place after ∼11.9 ka, where silt/clay ranges from 0.68 to 1.0, with a mean of ∼0.82. Over this interval, particles in the clay-size fraction (<4.0 µm) generally exceeded 50% of the terrigenous fraction.

Overall, the record of TMAR is marked by a major transition from low to relatively high rates after 14.3 ka. From ∼18.1 to 14.3 ka, the average was ∼0.004 gm/cm^2^/yr, and TMAR never exceeded ∼0.006 gm/cm^2^/yr. The transition after ∼14.3 ka was initially marked by an abrupt two-fold increase in TMAR. At a finer scale, the TMAR record shows a stepwise change from ∼14.3 to 9.5 ka. The initial step, from ∼14.3 to 12.8 ka, was characterized by TMAR values ranging from ∼0.007 to 0.012 gm/cm^2^/yr. This period is followed by brief decline to lower values from ∼12.8 to 11.9 ka, when the average TMAR was ∼0.006 gm/cm^2^/yr. The second TMAR increase was after ∼11.9 ka, with a mean value of 0.012 gm/cm^2^/yr until ∼10.1 ka.

## Interpretation

### Dense forest and chemical weathering

At the end of the LGM, paleoclimate records from throughout Africa and at Lake Malawi suggest cooler temperatures and aridity [14], [50]–[51]. Although it might be expected that these environmental conditions would slow reaction rates and mineral transformation during the early deglacial period, this does not seem to be the case in northern Malawi. Throughout this phase, K/S is relatively high in our core record (Figure 3). Higher proportions of kaolinite, a clay mineral leached of mobile cations typically associated with soils of the humid tropics, suggests that chemical weathering was relatively intense. The increased delivery of heavily weathered clays may appear counterintuitive given the comparatively cool temperatures and aridity during this time as recorded in the same core based on TEX-86 and leaf wax δ^13^C, respectively [50]–[51]; however, the clay mineralogy record is similar to changes in vegetation during the early deglacial period.

At the end of the LGM, many studies have suggested that East African afromontane forest communities expanded to lower altitudes than at present because of cooler temperatures and low atmospheric CO_2_ [52]–[54]. This was also the case within the Lake Malawi watershed, where afromontane taxa, dominated by *Juniperus*, Ericaceae, and *Podocarpus*, were found in high percentages at the earliest part of our record beginning around 18.1 ka and lasting until ∼15.5 ka (Figure 3) [46]. Although it is unlikely that the afromontane forest reached the lakeshore, Ivory et al. [46] suggest that these normally high-altitude communities expanded down to ∼900 masl within several kilometers of the lakeshore. Dense forest communities must have been prevalent within the watershed, given the presence of taxa typical of the lower montane moist forest (*Olea* spp., *Myrica*). Furthermore, grass percentages during this interval are very low, the lowest of the record (Figure 3). In modern lake-floor sediment samples, grass percentages within Lake Malawi contribute ∼55% of the total pollen [25], supporting the idea of a much denser canopy cover and larger contribution of the arboreal taxa throughout the watershed and into the lowlands. This may suggest that the presence of dense vegetation on the landscape as a result of lowering of the afromontane belt resulted in higher concentrations of organic acids and increased soil moisture, counteracting any slowdown in weathering due to climate.

The first marked change in both the vegetation and sedimentary records began at 15.5 ka, a period which is coeval with the end of Heinrich Stadial 1 (H1) [55]. Within Africa and at Lake Malawi, this period was characterized by progressive warming [50]–[51]. During this phase from 15.5 to 14.3 ka, K/S reached the highest values of the record (Figure 3). This suggests that, as in the previous phases of afromontane forest dominance, chemical weathering within the northern Malawi watershed remained relatively intense. Although some wetting occurs in East Africa during this post-H1 period, organic geochemical analysis from leaf wax δ^13^C implies that Malawi remained relatively arid with respect to modern at this time, despite indications of warming [50]–[51]. Once again, although chemical weathering reactions would be expected to slow during an arid period, intense weathering continued in the northern Lake Malawi watershed.

Following H1, all of the principal afromontane arboreal taxa began to decline [46]. This decline has been attributed to increasing temperatures which forced afromontane forest to retreat to higher elevations [25]. However, the transition was stepwise. Although the principal afromontane forest taxa that were dominant just following the LGM (Ericaceae, *Juniperus*, and *Podocarpus*) began to decline, from 15.5 to 14.3 ka, the dense lower montane moist forest taxa, such as *Myrica* and *Olea*, known pioneer taxa, continued to expand [46]. The presence of dense forest in the lowlands is the best explanation for the continued high values of K/S at this time.

Throughout the early deglacial period, minimum values of TMAR suggest that erosion was low (Figure 3). Johnson and McCave [20] suggest that TMAR in the northern basin of Lake Malawi is indicative of moisture, and therefore low values would suggest a reduced riverine transport. However, our dataset makes clear that a more likely mechanism for abating the flushing of weathered parent material from the landscape is the presence of dense lowland forests. During the LGM, a slight lake-level regression at Malawi of ∼40 to 100 m would have decreased the distance from the mouth of the Songwe-Kiriwa River and other important drainages to the core site [56]. A basinward progradation of the delta during a lake-level lowstand might be expected to show an increase in the amount of terrigenous material deposited; however, we observe the opposite. Thus, despite noted cooler temperature and aridity relative to modern, intense weathering and low erosion are observed over this period by K/S and TMAR. This regime strongly suggests that dense moist forest in the watershed had a strong control on clay transformation and delivery of terrigenous siliciclastic material to the basin.

*Transition to the Holocene (14.3 to 11.8 ka)*

At 14.3 ka, a transitional period began that was coeval with the onset of the Bolling-Allerod (BA) [57]. In contrast with the predominantly silty detritus in our record lower in the core, a broad range of grain sizes produces highly variable values of silt/clay (1.8 to 0.6) during this time (Figure 3). Over this interval, lake levels were highly variable. Evidence from diatom assemblages implies high-frequency changes on the order of 50 to 100 m [56]. Sand is present in the samples from this interval, but only in significant percentages in one sample. However, there is clear evidence of turbidity flows here as early as ∼13.2 ka (Figure 2). The presence of time-equivalent turbidites has also been described in other cores in northern Lake Malawi (“homogenites” of Barry et al. [58]). Thus, the variable silt content is expected to be caused by a change in sub-lacustrine depositional processes and energy. Thin, dominantly fine-grained turbidites may have been produced by wave remobilization of distal deltaic sediments, which move downslope at low velocity under the force of gravity as benthic nepheloid plumes [20].

At this time, the moist montane forest taxa became less abundant in the lowlands, and the afromontane forest as a biome reached very low percentages values, suggesting trees may have retreated to higher elevations by this time (Figure 3). Within the lowlands, however, arboreal taxa of the tropical seasonal forest began to increase in abundance. Miombo woodland abundances began increasing as well; however, the response time is much slower than the other arboreal taxa and values remained relatively low. Although the lowland closed-canopy tropical seasonal forest began expanding at this time, the percentages remained at <10%, values which are not significantly larger than those observed today, suggesting that dense forests were restricted largely to riparian corridors [25]. Ivory et al. [46] attribute this rise of lowland dense forest to the higher temperatures and reinforced monsoon recorded at the time in the basin. The lack of other arboreal vegetation on the landscape away from river banks in the lowlands indicates a marked change of physiognomy from the previous phase following the LGM to more open vegetation. This openness in the lowlands is reflected in the high percentages of grass pollen during this phase (Figure 3).

Within the basin, the increase in grass is tracked by rising TMAR values in core MAL05-2A (Figure 3). The increase in terrigenous material as the landscape opens supports the argument that TMAR represents erosion from the hinterland into the basin when vegetation is open and forests are scarce. This suggests that the relatively open character of vegetation caused by the retreat of montane forests prevented water storage on the landscape and promoted flushing of clastic material into the basin.

The presence of turbidites makes the clay record in this interval more complex. From 14.3 to 12.7 ka, an overall declining trend in K/S is observed, followed by an abrupt increase at 12.7 to 12.3 ka. Dense tropical seasonal forest and miombo woodland continued to expand in the lowlands at Lake Malawi between 13 and 11.7 ka, and tropical seasonal forest reached its maximum extent at the end of this interval (Figure 3). The beginning of this zone is coeval with the inception of the Younger Dryas (YD) in the Northern Hemisphere [59]. Although many other sites in East Africa record a retreat or slowdown of deglacial forest expansion during this time due to increased aridity, the dense lowland forest became more prevalent at Lake Malawi [60]-[64].

This indication of dense forest in conjunction with higher K/S values could possibly suggest a return to more intense chemical weathering beginning around 12.7 ka. However, independent particle size evidence, which shows elevated silt/clay and minor sand in the core during the period from 12.7 to 12.3 ka, suggests instead that the elevated K/S may be related to minor reworking by turbidites. Therefore, we interpret the multiple sedimentary indicators from this interval to be influenced by mass wasting. This may be the result of flushing from enhanced precipitation promoting sediment transport (and hyperpycnal sub-lacustrine flows) rather than chemical weathering.

### Early Holocene open woodlands

Although records from farther north in tropical and subtropical Africa show an abrupt resumption of the African monsoon and significant wetting during the early Holocene, including at Lake Rukwa (∼200 km north of Lake Malawi), the situation at Lake Malawi is slightly different [14], [65]. Organic geochemical studies of δ^13^C agree that the watershed was wetter than during the late Pleistocene; however, a ∼100 m lake-level regression occurred at this time, suggesting a change in hydrology [66]–[67]. Pollen analysis on a core from Lake Masoko, a small maar lake within the Malawi watershed, suggests that the expansion of open miombo woodlands indicates high summer rainfall, but heightened rainfall seasonality [47]. This conclusion is in agreement with Zr:Ti and biomarker studies from Lake Malawi, which demonstrate wind direction and air mass changes at this time consistent with major reorganization of tropical circulation and a more northerly ITCZ than during the late Pleistocene [11], [68].

At this time, total abundance of clays was very high, resulting in a net increase in all dominant clay minerals; however, K/S reached a minimum (Figure 3). Additionally, for the first time in this record, illite plus chlorite reached higher abundances than kaolinite (Figure 3). The dominance of detrital clays retaining mobile cations suggests a very strong decrease in the intensity of chemical weathering beginning at 11.8 ka. Furthermore, the importance of illite, a clay mineral that is only present in low abundances in the modern lake today, is suggestive of increasing physical weathering and reworking during the early Holocene despite increased wetness [48]. In addition, TMAR during this interval reaches maximum values. The agreement of these two independent datasets seems to indicate peak erosion from 11.8 to 10 ka.

This transition in clay mineralogy at 11.8 ka is coeval with the opening of the lowland vegetation (Figure 3). At 11.8 ka, near the onset of the Holocene, an abrupt transition occurred from denser lowland forests to more open miombo woodland in the watershed (Figure 3). Additionally, for the first time in the record, afromontane forest tree pollen is nearly absent. Given the high abundance of woodland and grasses, it is likely that this early Holocene transition represented that largest change in vegetation physiognomy in Malawi during the studied interval.

This increase in erosion was likely caused by the more intense summer rains of the African monsoon and the open character vegetation, conditioning the landscape for the flushing of physically weathered material into the basin. Cecil and Edgar [69] noted a similar relationship between greater coarse siliciclastic sediment transport and strongly seasonal rainfall.

## Discussion

Traditional models for the tropics suggest strong climatic controls such as precipitation and temperature on chemical and physical weathering and erosion [44], [70]. However, our record from Lake Malawi suggests a more indirect role of climate. During the early part of the last deglaciation, climate within the watershed varied dramatically in mean state and variability, including cooler temperatures and relative aridity from 18.1 to 14.5 ka. This was followed by successive wetting and warming from 14.5 to 12.5 ka, a return to slightly drier conditions with much reduced rainfall seasonality during the YD, and finally an increase in rainfall seasonality at the time of the resumption of the African monsoon at the early Holocene. However, despite the changes in climate, both highland and lowland forests were common in the watershed throughout this time until 11.8 ka. Although changes in rainfall and temperature lead to compositional vegetation changes during this period as high-altitude forest was replaced successively by lowland forest, the physiognomy of the landscape, with dominance of arboreal taxa, remained until the early Holocene. Similarly, although chemical weathering should be more intense during the moister periods, instead we see relative stability in the clay minerals with higher values of kaolinite throughout this period regardless of climatic regime. During the early Holocene, the physiognomic change to more open woodland is coeval with higher mean annual precipitation because of heightened seasonality following the resumption of the African monsoon. It might be expected that this high temperature and high rainfall should have resulted in intense chemical weathering, but the available data do not support this idea. At this time, kaolinite decreases, and high abundance of illite suggests not only less-intense chemical weathering, but higher physical weathering and transport of less-weathered micas into the basin.

The strong, direct control of vegetation on mineral transformation and erosion is not surprising for several reasons as many processes mediated by plants influence chemical and physical breakdown of bedrock. First, although heat and moisture are necessary for driving chemical breakdown of aluminosilicate rocks, organic acids, particularly low-weight organic acids such as those produced by plants and mycorrhizae associated with plant roots, are essential for biological mediation of weathering in soils [71]–[72]. These acids not only provide H+ for the transformation of primary materials, but also act as ligands that form strong complexes with trivalent cations such as Al^3+^ and Fe^3+^ [6]. This function of organic acids has the potential to dramatically intensify weathering rates by increasing the solubility of minerals containing these elements. Second, root networks, particularly in dense, old-growth forests, are critical for the mechanical breakdown of bedrock [73]. Furthermore, the removal of trees results in an increased response of erosion rates and closer control of abiotic erosion mechanisms as seen in our record.

Perhaps the most important biotic control on weathering is the role of forests and large forest trees in regulating the hydrologic cycle locally within a watershed and at the scale of a soil [74]. In dense forests, canopies intercept rainfall. During intense monsoon rains, this function acts to reduce runoff and increase storage on the landscape, increasing the residence time of moisture available for chemical reactions in soils. Additionally, evapotranspiration within a forest often creates a microclimate that results in higher local precipitation over dark, dense trees as well as more stored water on the landscape and cooler temperatures from evaporation [75]. More importantly however, evapotranspiration controlled by forest trees limits the drainage depth within the weathering profile of a soil, reducing physical weathering processes and allowing time for chemical alteration [76]. Vegetation transitions, as we see in the Malawi record, however, are key as long-lived dense forests, such as those found in the Congo Basin or in the Amazon exhibit very little chemical weathering [77]–[78]. Early stages of succession, such as the period of intense chemical weathering observed during the deglaciation following the retreat of the afromontane forest as lowland forest colonized the lake shore, are in fact typically the period of most intense chemical weathering as large trees colonize previously unweathered substrate [79]–[80].

TMAR values show a positive correlation with grass abundances throughout our record. Although TMAR has been interpreted as an indication of moisture availability, we instead suggest that delivery of terrigenous material to the basin is strongly regulated by vegetation on the landscape. This means that more open, grassy woodlands are less effective at trapping material on the landscape leading to greater erosion and deposition of terrigenous material than when forests are dominant in the lowlands [12], [81]. This first order control of erosion on the landscape is in agreement with cosmogenic nuclide studies in the Alps by Vanacker et al. [82] who show exponential increases in denudation rates due to modern land use change. Although this suggests rapid landscape conversion once forest is gone, this study, as well as that of Vanacker et al. [82], suggests that once forest returns, stabilization is possible as denudation rates return to background levels. Reducing the residence time of water on the landscape likewise influences chemical weathering intensity, and we note that the inverse correlation of K/S with TMAR and grass percentages, which appears to support this hypothesis.

In our analysis of core records from Lake Malawi, we have observed coarser sediments (high silt/clay or % sand) around the inception of lake-level highstands, which also show high ratios of trees to herb pollen. Several factors may influence this particle size trend. For example, storage release (i.e., flushing) of coarser sediment from low-to-moderate gradient flexural and axial margins concomitant with transgression likely helps to explain some of the variability we have observed. Additionally, increased fluvial discharge implied by the presence of afromontane forest is consistent with what is known about the efficacy of coarse siliciclastic transport [34]. Another potential influence relates to the shortening of fluvial transport networks associated with the higher base level (i.e., less reduction of siliciclastic particle size by transport abrasion and downstream fining), but because fining upward patterns are characteristic of the highstand strata we have examined, the absolute influence of this mechanism is not well known. By contrast, during lowstand intervals such as the early Holocene, when dry season length was long, fluvial networks lengthen and appear to generate finer siliciclastic detritus. Physical weathering of parent rocks and soils, captured in the clay mineral record, may serve as a feedback on reducing particle size during these intervals. Indeed, the lack of forest cover to intercept rainfall during these intervals may also serve to help flush fine sediment from hillslopes and floodplains into river channels. Variability in depositional processes can complicate the interpretation of silt/clay, which is why evidence of mass wasting is critical to identify in the lithostratigraphy. For example, the transition from lowstand to highstand in rift lakes occurs rapidly (10^2^ to 10^5^ yrs) [83], and as such flexural margins can become prone to gravity flows as pore pressures change and slopes destabilize. Therefore, our study employed particle size and stratigraphic data in support of pollen, clay minerals, and TMAR to provide the most rigorous assessment of weathering and erosion patterns for the deglacial period yet developed for this region of East Africa.

## Conclusions

Vegetation composition and structure at Lake Malawi and elsewhere does not unequivocally track simple precipitation amount [28], [46]–[47]. Our record attests to a strong negative relationship between increased rainfall seasonality and vegetation density that results in a specific depositional signature within the lake. When dense forests occupy the watershed, chemical weathering is intense and erosion is low. Leaching within soils leads to generation of highly altered clay minerals like kaolinite. However, during times of open vegetation, chemical weathering is less intense, but erosion is increased. Smectite dominates during these periods, but flashy precipitation on the open landscape leads to flushing of siliciclastics into the lake and thus high relative TMAR.

Finally, these results have important implications for better understanding how weathering and erosion may change in the future. Although little work has been done to quantify potential future alterations in weathering, most models do not take ecosystem change related to climatic change into account, leading to significant potential biases. Only in the last few years has vegetation been added to projections of soil weathering, thus leading to the suggestion of high sensitivity of weathering to future climate states [84]–[86]. Although paleostudies hint at the role of biological mediation in weathering over long time scales, no other studies have used paleo-records to quantify the effects of vegetation on weathering processes in southeast Africa [7]. This study points to the importance of vegetation for mediating weathering in the past; however, more and longer records are needed in order to better quantify this effect over a larger range of climatic and vegetation variability in watersheds throughout the tropics.

## Supporting Information

Table S1 **Pollen counts from core MAL05-2A.**
(XLSX)

Table S2 **Particle size and clay mineralogy from core MAL05-2A.**
(XLSX)

Methods S1 **Materials and methods details of particle size and x-ray diffraction analysis.**
(DOC)
