# Supplementary material for: Vegetation Controls on Weathering Intensity during the Last Deglacial Transition in Southeast Africa
Source: PLoS One. 2014 Nov 18;9(11):e112855. doi: 10.1371/journal.pone.0112855 (PMC4236122; doi:10.1371/journal.pone.0112855)
Supplement: Methods S1 — Materials and methods details of particle size and x-ray diffraction analysis. (DOC) [file pone.0112855.s003.doc]

Supplement Materials

Particle Size Analysis

Parameter testing indicated that absorption and obscuration, perhaps the two most important variables for size analysis by laser-diffraction (Sperazza et al., 2004), produced low residual values when optimized at ~0.6 and 15-20%, respectively. Samples were stirred at 3425 rpm and sonicated at 85% for 1 minute prior to and during measurements. Each sample was measured four times, and we present our data as an average ratio of silt to clay. Prior to analysis, samples were chemically treated in an 85°F immersion bath to remove the organic matter (via multiple 30% H_2_O_2_ digestions) and opal (via multiple 2N NaOH digestions) that constituted a significant proportion of the bulk sediment. Samples were also treated with 20% HCl at room temperature to remove any traces of authigenic carbonate. Visual examination of smear slides confirmed the removal of biogenic components, and we estimate that refractory biochemical materials highly resistant to chemical attack constituted <1.0% of the sub-samples. To avoid flocculation of clays, individual aliquots of isolated terrigenous sediment were suspended in sodium hexametaphosphate (NaPO_3_)_6_ and shaken on a wrist-action shaker for several days prior to analysis.

Clay Mineralogy

Samples were micronized and spiked with a corundum standard, then scanned from 5 to 65 degrees 2Θ (0.02 degree step) with a Scintag X-1 x-ray diffraction (XRD) system. Diffraction patterns were analyzed using RockJock (Eberl, 2003), which determines the weight percent of pre-selected minerals using a peak fitting routine and a library of mineral standard patterns (including amorphous or poorly crystalline components common in lake sediments, such as kerogen and diatoms). Assuming minimal deviation from standard patterns and proper sample preparation, the measurement error ascribed to RockJock is ±5%. Because clay minerals are a primary focus of this study, we also assessed several samples from each lithostratigraphic unit in the MAL05-2A deglacial sequence using qualitative XRD methods (Moore and Reynolds, 1989). Oriented mounts were prepared using the modified filter-peel method of Pollastro (1982), and clay minerals were identified using the guidelines of Moore and Reynolds (1989). This step also served to validate RockJock inputs. Given shallow burial depths (<10 m) and the likely hydrochemistry that prevailed in Lake Malawi’s northern basin during the deglacial interval (Stone et al., 2011), we assumed that diagenetic formation of clays is minimal. The validity of this assumption is supported by the scanning electron microscopy findings of Kalindekafe et al. (1996), which indicated a detrital origin for Lake Malawi’s modern and Holocene clays on the basis of grain shape and abrasion patterns. Furthermore, Scholz et al. (2007) demonstrated that over the past ~60 kyr, water volume changes associated with climate-driven lake level fluctuations were minimal at Malawi. Thus, a dominantly detrital origin for clays deposited during the deglacial transition is consistent with what is known from independent paleoclimate evidence.

References

Eberl, D.D. (2003). User's guide to RockJock -- A program for determining quantitative mineralogy from powder X-ray diffraction data: U.S. Geological Survey Open-File Report 2003-78, 47 p.

Kalindekafe, L.N., Dolozi, M.B., and Yuretich, R. (1996). Distribution and origin of clay minerals in the sediments of Lake Malawi, in Johnson, T.C., and Odada, E., eds., The Limnology, Climatology and Paleoclimatology of the East African Lakes: Amsterdam, Gordon and Breach, p. 443-460.

Pollastro, R. M. (1982). A recommended procedure for the preparation of oriented clay–mineral specimens for X-ray diffraction analysis. U.S. Geological Survey Open File Report, pp. 71–82.

Scholz, C.A., Johnson, T.C., Cohen, A.S., King, J., Peck, J., Overpeck, J., Talbot, M., Brown, E., Kalindekafe, L., Amoako, P., Lyons, R., Shanahan, T., Castañeda, I., Heil, C., Forman, S., McHargue, L., Beuning, K., Gomez, J., and Pierson, J. (2007). East African megadroughts between 135 and 75 thousand years ago and bearing on early-modern human origins. Proceedings of the National Academy of Sciences, 104, 16416–16421.

Sperazza, M., Moore, J. N., and Hendrix, M. S. (2004). High-Resolution Particle Size Analysis of Naturally Occurring Very Fine-Grained Sediment Through Laser Diffractometry. Journal of Sedimentary Research, 74(5), 736-743.

Stone, J.R., Westover, K.S., and Cohen, A.S. (2011). Late Pleistocene paleohydrography and diatom paleoecology of the central basin of Lake Malawi, Africa. Palaeogeography, Palaeoclimatology, Palaeoecology, 303, 51-70

Any use of trade, product, or firm names is for descriptive purposes only and does not imply endorsement by the U.S. Government.
